# Proof of Concept: Development of Snow Liquid Water Content Profiler Using CS650 Reflectometers at Caribou, ME, USA

**DOI:** 10.3390/s17030647

**Published:** 2017-03-21

**Authors:** Carlos L. Pérez Díaz, Jonathan Muñoz, Tarendra Lakhankar, Reza Khanbilvardi, Peter Romanov

**Affiliations:** 1 National Oceanic and Atmospheric Administration-Cooperative Remote Sensing Science and Technology (NOAA-CREST) Center, The City College of New York, New York, NY 10031, USA; tlakhankar@ccny.cuny.edu (T.L.); rk@ce.ccny.cuny.edu (R.K.); 2 Department of Civil Engineering and Surveying, University of Puerto Rico, Mayagüez, PR 00681, USA; jonathan.munoz@upr.edu; 3 National Oceanic and Atmospheric Administration-National Environmental Satellite, Data, and Information Service (NOAA-NESDIS), Camp Springs, MD 20740, USA; peter.romanov@noaa.gov

**Keywords:** snow, wet snow, snow wetness, snow liquid water content

## Abstract

The quantity of liquid water in the snowpack defines its wetness. The temporal evolution of snow wetness’s plays a significant role in wet-snow avalanche prediction, meltwater release, and water availability estimations and assessments within a river basin. However, it remains a difficult task and a demanding issue to measure the snowpack’s liquid water content (LWC) and its temporal evolution with conventional in situ techniques. We propose an approach based on the use of time-domain reflectometry (TDR) and CS650 soil water content reflectometers to measure the snowpack’s LWC and temperature profiles. For this purpose, we created an easily-applicable, low-cost, automated, and continuous LWC profiling instrument using reflectometers at the Cooperative Remote Sensing Science and Technology Center-Snow Analysis and Field Experiment (CREST-SAFE) in Caribou, ME, USA, and tested it during the snow melt period (February–April) immediately after installation in 2014. Snow Thermal Model (SNTHERM) LWC simulations forced with CREST-SAFE meteorological data were used to evaluate the accuracy of the instrument. Results showed overall good agreement, but clearly indicated inaccuracy under wet snow conditions. For this reason, we present two (for dry and wet snow) statistical relationships between snow LWC and dielectric permittivity similar to Topp’s equation for the LWC of mineral soils. These equations were validated using CREST-SAFE in situ data from winter 2015. Results displayed high agreement when compared to LWC estimates obtained using empirical formulas developed in previous studies, and minor improvement over wet snow LWC estimates. Additionally, the equations seemed to be able to capture the snowpack state (i.e., onset of melt, medium, and maximum saturation). Lastly, field test results show advantages, such as: automated, continuous measurements, the temperature profiling of the snowpack, and the possible categorization of its state. However, future work should focus on improving the instrument’s capability to measure the snowpack’s LWC profile by properly calibrating it with in situ LWC measurements. Acceptable validation agreement indicates that the developed snow LWC, temperature, and wetness profiler offers a promising new tool for snow hydrology research.

## 1. Introduction

Seasonal snow is an influential reservoir constituent in the hydrological cycle that discharges temporarily stored freshwater to the forelands [1,2]. Downstream water suppliers (e.g., Western USA, the Rhine River in Europe, the Canadian prairies, etc.) [3] are greatly reliant on snow meltwater discharge from the alpine head watersheds to supply potable and irrigation water. An important snow parameter, the snow water equivalent (SWE) is commonly known as the amount of water contained within the snowpack, or the depth of water that would theoretically result if the entire snowpack melted instantaneously [4,5]. However, this measurement does not deliver information on the condition of melting snow. On the other hand, the liquid water content (LWC), *θ_w_*, of the snowpack describes its snow wetness. Snow wetness is used as an indicator of snow melt and snow instability [6]. An increase in the liquid water (wetness) of the snowpack leads to an onset of meltwater runoff within a catchment. This type of information is relevant for flood predictions during intense melting due to rain-on-snow events combined with warm air temperatures [7,8]. Quantitative and temporal meltwater delivery predictions are often required by water resources engineers and decision makers in the water management field to deal with: reservoir management and hydropower generation [3,9], and catchment runoff and flood forecasts [10,11,12]. Additionally, information on the LWC and wetness of the snowpack is essential for wet-snow avalanche forecasting, because the permeating water in the snowpack dampens its mechanical strength and creates instability [13,14,15].

Generally, snow wetness is quite difficult to measure in situ. Furthermore, to account for the spatiotemporal evolution of meltwater runoff and snow instability, non-destructive and continuous snow LWC monitoring is necessary because changes in the LWC can rapidly alter various snowpack properties and its meltwater outflow [15,16]. Moreover, these processes are non-linear and, as such, difficult to detect or forecast, and the most common type of measurement, manual snow wetness observations in snow pits, only provides a rough estimate and is based on a wetness index [17].

Techel and Pielmeier [18] and Boyne and Fisk [19] provide a review of numerous in situ snow wetness measurements. The majority of these in situ snow wetness measurement techniques are based on dilution, centrifugal, dielectric, and calorimetric measurement methods. Common instruments used to measure the permittivity of wet snow are: the Finnish Snow Fork [20] and the Denoth meter [21,22]. However, these measurement techniques are known to be destructive, time-consuming, and need to be executed at accessible sites. Other less invasive and, consequently, non-destructive in situ methods that have been employed include: time-domain reflectometry (TDR) [23,24,25,26,27,28], the Snow Pack Analyser (SPA) [29], and the upward-looking frequency modulated wave (upFMCW) and ground-penetrating radar (upGPR) systems [30,31,32]. The latter being unreasonably expensive (order of magnitude of 10, when compared to TDR instrument setups), difficult to install (large antennas at the lower frequencies, heavier than TDR reflectometers by an order of magnitude of 100), and requires specific, additional post-processing.

The aim of this study is to: (1) test the capability of the CS650 soil water content reflectometer to provide reliable snow LWC measurements and (2) provide a non-destructive, non-invasive, low-cost, automated, and continuous alternative to in situ snow LWC measurements at different snowpack heights above the soil surface by creating a snow wetness profiler (SWP) setup using TDR and a series of CS650 water content reflectometers. The determination of LWC using TDR is based on the relationship that exists between the relative complex dielectric constant of the medium and its water content. The first use of TDR to determine LWC was executed in soils by Topp et al. [33,34,35]. Later, time-domain reflectometry was proven to be a technique that can be used to indirectly measure the in situ LWC of snow (and monitor snowmelt percolation in the snowpack) by Stein and Kane [24]. They presented the first application of TDR to the measurement of snow density and LWC. However, they only showed the signal, but did not make any calibrations. Stein and Kane only explained how to use TDR to obtain the LWC of snow by means of its relative complex dielectric constant and density. Later, Schneebeli and Davis [36] calibrated the dielectric constant and LWC of snow for a limited number of values. Stein et al. [25] and Schneebeli et al. [37] expanded on the practice of the TDR technique in snow, and developed relationships between its relative complex dielectric constant, density, and LWC by conducting separate field campaigns and, in the case of Schneebeli et al. [37], laboratory experiments using time-domain reflectometers (model Tektronix 1502B (Melrose, MA, USA)). Concurrently, Lundberg [26] performed a laboratory comparison of the TDR technique with the dilution method, and demonstrated that TDR has the potential to register variations in snow liquid water content down to 1–2 vol. % by fitting an empirical model to seasonal snow of a higher density (350 kg/m^3^). Moreover, Lundberg concluded that continuous registration of snowpack wetness with acceptable spatial resolution (approximately 5 cm) was possible to achieve with several sets of probes—mounted with 3 cm vertical and 5 cm horizontal spacing—combined with a multiplexer and a storage unit to record the data. Additionally, a more recent study by Waldner et al. [38] makes use of literature on the dielectric properties of snow by Looyenga [39], Tiuri et al. [40], and Frolov and Macharet [41] to calibrate two newly developed TDR sensors to estimate snow wetness and density. Consequently, Waldner et al. [42] make use of the TDR equipment described by Schneebeli et al. [37] to derive the dielectric permittivity of snow (and its LWC) using the algorithm described in his previous study [38]. Ultimately, the efforts by Waldner et al. [38,42] demonstrated new instrument setups that make use of TDR to estimate snow density and LWC.

To our knowledge, there is no existing literature on the usage of the CS650 TDR sensor to provide snow LWC measurements. Furthermore, most of the snow TDR studies mentioned previously have been conducted manually, not in automated, continuous fashion, nor has an in situ season-long, field-withstanding instrument that provides the LWC and temperature profiling (at different depths above the soil surface) of the snowpack simultaneously been developed. Therefore, the SWP will be a novel contribution as an alternative to execute year-round, automated, undisturbed in situ snow LWC measurements, and provide insight on the snowpack state (e.g., dry, moist, wet) at different snowpack heights above the soil surface. The SWP was developed using two arrays of low-cost (approximately $400 per array; will depend on quantity of reflectometers) and -power consuming, easily-assembled (reflectometers come ready to be used, no need for prior setup) and –installed (reflectometer instructions provide all the information needed for cable connection to datalogger) moisture reflectometers. SWP observations are automated and continuous.

Snow Thermal Model (SNTHERM) LWC simulations forced with in situ meteorological data were used to evaluate the accuracy of the instrument due to the lack of LWC in situ observations at the study site (Section 2). In order to cross-validate the accuracy of SNTHERM LWC simulations, these were compared to LWC estimates obtained using empirical formulas developed in previous studies by Topp et al. [33], Denoth et al. [21], and Tiuri et al. [40]. Additional contributions to snow TDR and hydrology are presented in the form of the development of two (for dry and wet snow) statistical relationships between snow LWC and dielectric permittivity of similar nature to Topp’s equation for the LWC of mineral soils.

Section 2 provides a brief description of the study area and an overview of the Cooperative Remote Sensing Science and Technology Center-Snow Analysis and Field Experiment (CREST-SAFE) station. In Section 3, an outline of the SWP setup and the methodology are discussed. Section 4 and Section 5 illustrate the results and discussion, respectively. Section 6 provides the conclusions and future work.

## 2. Study Area and the CREST-SAFE Station

**Figure 1:**
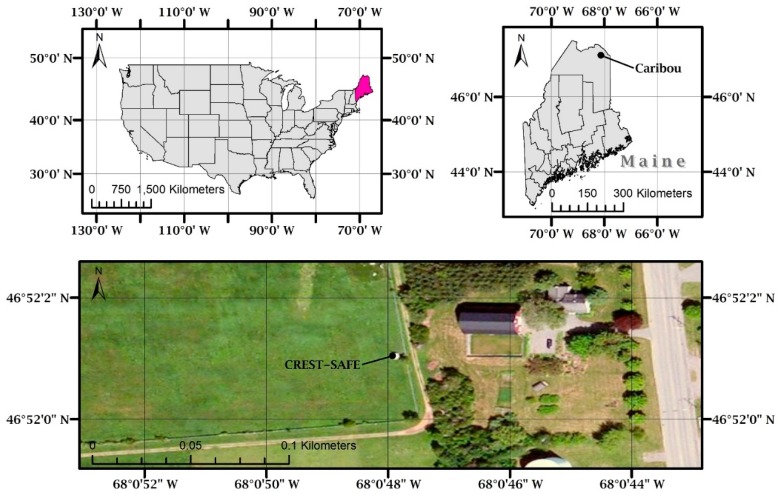
CREST-SAFE location near the National Weather Service Regional Forecast Office and within the Caribou Municipal Airport premises in Caribou, ME, USA.

In situ observations were performed in the county of Caribou in the state of Maine located in the Northeast of the United States of America. The coordinates for the study area are 46°55′ N, 68°01′ W (Figure 1). The site is apt and suitable for snow research, given how it is covered in snow from late November to early April. Average seasonal snowfall is 2.8 m, with a record high of 5 m. Seasonal snow accumulation typically reaches its maximum (around 50–60 cm) by late February or the beginning of March. The region is categorized as gentle rolling terrain with low-rounded mountains that elevate from 120–300 m, and, approximately, half of the land adjacent to the study area is agricultural. The rest is considered to be spruce fir forests and maple beech birch. Forest fraction increases from southeast to northwest in the region. Four active United States Geological Survey (USGS) gauges record streamflow information of the Aroostook River, as well as other smaller rivers and streams. Snowmelt water presents the majority of the yearly runoff (daily averages from as low as 6 (January/February) to 57 (April) m^3^·s^−1^, monthly averages from as low as 1.5 (January/February) to 16 (April) m^3^·s^−1^, and an annual average of ~ 4 m^3^·s^−1^) (https://nwis.waterdata.usgs.gov) to local rivers.

The Field Snow Research Station (also referred to as Snow Analysis and Field Experiment, SAFE) is operated by the NOAA Cooperative Remote Sensing and Technology Center (CREST) in the City University of New York (CUNY). The field station is located within the premises of the Caribou Municipal Airport (46°52′59′′ N, 68°01′07′′ W) and in close proximity to the National Weather Service (NWS) Regional Forecast Office. The station was established in 2010 to support studies in snow physics and snow remote sensing. The surrounding area in direct contact with the instruments at the station is covered in grassland.

The station tallies a total of twenty-two (22) instruments that provide continuous and automated all-year round measurements of the physical characteristics of snow, soil temperature down to 20 cm into the ground, and all basic meteorological parameters. The list of measured snow pack physical properties includes: snow depth, SWE, snowfall, and the snowpack temperature profile. Two station web cameras provide real time images of the site [43,44]. The observation of snowpack physical properties is complemented with snowpack radiative measurements in the infrared and microwave spectral bands. Additional instruments include: a SWE gamma sensor and the developed snow wetness profiler discussed in this study. Table 1 offers a brief description of each instrument’s measured/observed parameter, accuracy and temporal sampling interval. Access to all ground-based observation records (i.e., snowpack physical properties, snow stratigraphy, and microwave radiometer data) can be obtained in the CREST-SAFE website [45].

Note: In situ snow depth, SWE, and near-surface air temperature observations from CREST-SAFE will be used in Section 4 and Section 5 to assist the explanation and discussion of the results of this study. CREST-SAFE snow depth and SWE observations will be used to calculate bulk snow density as the ratio between the SWE and snow depth. Bulk snow density will be used to compute LWC estimates in Section 4.

## 3. Methodology and Materials

### 3.1. Time-Domain Reflectometry

Time domain reflectometry is a highly accurate and automatable method for the determination of the LWC of porous media and its electrical conductivity [28]. LWC is inferred from the dielectric permittivity of the medium, whereas electrical conductivity is inferred from TDR signal attenuation. In a fairly simple approach, empirical and dielectric mixing models are used to relate a medium’s LWC, dielectric permittivity, and density. In some cases, the relationship between LWC and dielectric permittivity requires individual calibration when dealing with non-mineral (e.g., clay and organic matter, etc.) soil media or snow. Numerous TDR probe configurations provide users with site- and media- specific options. Hence, continuous developments in TDR technology and in other dielectric methods offer the promise for less expensive and more accurate tools for the electrical determination of LWC.

Time domain reflectometry is related to the measurement of the relative complex dielectric constant, which is a component of the capacitance [24]. The capacitance is a constant of proportionality that relates the potential difference between conductors to the amount of equal, but opposite electric charges in each of them. Capacitance is quite dependent on the geometry of the two conductors and the relative complex dielectric constant [46]. However, the relative complex dielectric constant (Equation (1)) is variable for most materials; it has a real and an imaginary part; both frequency dependent [33]:

(1) $$K\;=\;K^{\prime }+j\left\{\left(\sigma_{dc}/\omega \varepsilon_{0}\right)\;K\prime \prime \right\},$$

where K complex dielectric constant; $K^{\prime }$ real dielectric constant; K″ dielectric loss; $\sigma_{dc}$ conductivity; ω angular frequency; $\varepsilon_{0}$ permittivity of free space; and j = (−1)^1/2^.

Thus, the complex dielectric constant of a material can be determined from the propagation of a pulse along a transmission line. The velocity of a pulse ($v_{p}$) along a transmission line is given by:

(2) $$v_{p}=\;L_{r}/t,$$

where $L_{r}$, is the physical length of the transmission line (or length of the probe) and t is the time of propagation. Furthermore, distributed circuit analysis dictates that at high frequencies, and for non-magnetic materials [27]:

(3) $$v_{p}=\;c/K^{1/2},$$

The combination of both equations yields:

(4) $$K={\left(ct/L_{r}\right)}^{2},$$

where $L_{r}$ is the length of the line set by the user, c is the velocity of an electromagnetic wave in free space, and t is determined using the time domain reflectometer. Time domain reflectometry measures both the real and imaginary parts of the complex dielectric constant (as shown in Equation (1)). As such, the term “apparent dielectric constant” $\left(K_{a}\right)$ is sometimes used. However, for low loss materials (i.e., snow), $K=K^{\prime }$ and hence, $K_{a}=K^{\prime }$ [24,25,26,46]. Therefore, in this paper the dielectric constant refers only to the real part.

In short, a TDR measurement unit consists of a pulse generator (generating a step pulse, which is being transformed to a lower frequency), a registration unit (an oscilloscope for commercially available TDR units) and one or several sets of rods (probes) mounted parallel in the studied medium. The dielectric constant (electrical permittivity) can be determined from the velocity of propagation of an electromagnetic wave through a medium. The generated pulse is reflected when it reaches the end of the rods. The travel time of the reflected pulse is a function of the dielectric constant of the surrounding medium. Because the difference in dielectric constant between water (K_w_~80) and ice (K_I_~3) is significant at the 1 MHz to 1 GHz range, the dielectric constant is primarily a function of the liquid water content of the snow [26,38,39,40]. Additionally, density variations in the snow have some influence in dielectric constant measurements due to the difference between the dielectric constants of air (K_A_ = 1) and ice (K_I_~3) [26]. This is particularly true for wet snow because it is a mixture of ice crystals, liquid water, and air.

### 3.2. CS650 Time-Domain Reflectometer

The CS650 (Campbell Scientific, Logan, UT, USA) is a multi-parameter sensor that uses TDR to measure the liquid water content and electrical conductivity of soils and other porous media. Additionally, it measures the temperature of the medium via a thermistor in contact with one of the rods. It consists of two 30-cm-long (3.2 mm diameter and 3.2 cm spacing) stainless steel rods connected to a printed circuit board. The circuit board is encapsulated in epoxy, and a shielded cable is attached to it for datalogger connection. A five conductor cable including the drain or shield wire is used to provide power and ground as well as serial communication with the CS650. The CS650 is intended to communicate with SDI-12 recorders, including Campbell Scientific dataloggers [47]. The CS650 measures propagation time (converted to period), electrical conductivity and signal attenuation, and temperature [47]. Dielectric permittivity and liquid water content are then derived from these raw values. Period, electrical conductivity, and signal attenuation are converted to dielectric permittivity [47]. Liquid water content is obtained as a function of the medium’s dielectric permittivity with empirical formulas. This is discussed in Section 3.4.

Because the CS650 is a time-domain reflectometer, its fundamental principle is that the velocity of electromagnetic wave propagation along the probe rods is dependent on the dielectric permittivity of the material surrounding the rods, as discussed in Section 3.1. In order for the CS650 to perform a measurement, a differential emitter-coupled logic (ECL) oscillator on the circuit board is connected to the two parallel stainless steel rods. The differentially driven rods form an open-ended transmission line in which the wave propagation velocity is dependent upon the dielectric permittivity of the media surrounding the rods. An ECL oscillator state change is triggered by the return of a reflected signal from the end of one of the rods. Digital circuitry scales the high-speed oscillator output to an appropriate frequency for measurement. CS650 accuracy and precision for soil temperature, dielectric permittivity, and LWC measurements are presented in Table 2.

### 3.3. Snow Wetness Profiler Setup

**Figure 2:**
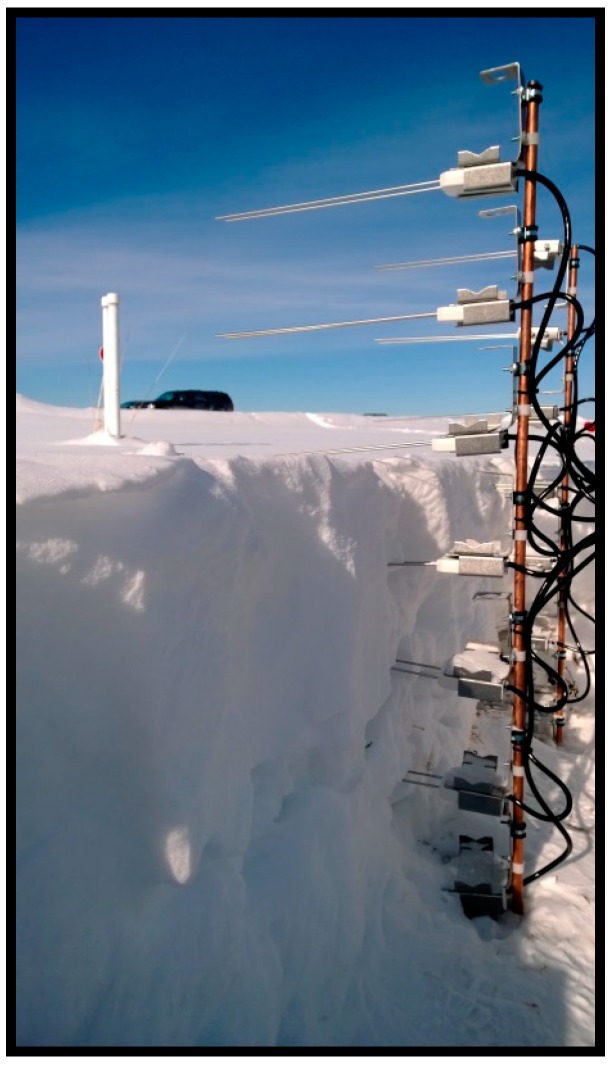
Snow Wetness Profiler at CREST-SAFE.

The SWP (Figure 2) was built with fourteen (14) CS650 soil water content reflectometers every 15 cm—all the way up to 90 cm—following, to some extent, the design by Lundberg [26]. The assembly consists of reflectometers attached to steel rods and is made up of two (7 reflectometers each) vertical profiles executing parallel measurements with a 1-h temporal resolution. The idea of having parallel observations is mainly to have backup measurements in case of instrument failure, but it also provides some spatial variability to the snow LWC measurements. The development of the SWP provides, aside from a cost-efficient, non-destructive way to measure the LWC of snow, the user with the capability to investigate the temporal variability of snow wetness throughout the winter. The SWP has also the capability of measuring the snowpack temperature, providing a vertically-distributed temperature profile.

The operational principle of the sensor is quite simple. When open to the air, the CS650 records the air permittivity, which is one (1). However, when in contact with a porous medium, the instrument records the permittivity (non-one number) of the medium, in this case snow. The instrument was installed on 6 February 2014. At the time, there were around 60 cm of snow in the ground at the station. The results shown in Section 4 comprise observations from (what are considered) two winters: 6 February 2014 to 22 April 2014 (from installation to when the snowpack melted in its entirety) and 24 November 2014 to 29 January 2015 (following winter until datalogger stopped recording; this is discussed in Section 4). It should be noted that the snowpack was disturbed during the installation process because some snow had to be removed in order to set up the SWP. The removal of the snow left the snowpack open to the air. While the specific day of installation was significantly cold (regularly conducive to a dry snowpack), this exposure might have altered the snowpack’s moisture. Nonetheless, all CS650 reflectometers were inserted into the snowpack during this process to make sure these were in contact with the snow, not the air. However, it is acknowledged that this issue could potentially lead to errors due to the presence of air bubbles between the probe rods and the snow, and cause additional snowmelt when the air is warmer than the snowpack. This is discussed in detail in Section 4 and Section 5.

Lastly, the CS650 information is stored on a datalogger (CR 3000) at CREST-SAFE. The CR 3000 was designed for stand-alone operation in harsh, remote environments. The orange Rx wire in the reflectometers can be used to communicate by means of RS-232 Tx/Rx. The A200 USB-to-Serial Module allows RS-232 serial communication between a computer and the CS650 by means of Campbell Scientific’s Device Configuration Utility (DevConfig) software.

### 3.4. Obtaining LWC from Dielectric Constant Measurements via Empirical Formulas

Campbell Scientific recommends the use of Topp’s equation (Equation (5)) to obtain LWC measurements as a function of the dielectric constant of the medium, as measured by the CS650 probes. Topp et al. [33] describe (empirically) the relationship between dielectric permittivity and LWC in mineral soils with a 3rd degree polynomial. With $\theta_{w}$ the liquid water content and K the dielectric permittivity of the soil, the equation presented by Topp et al. is:

(5) $$\theta_{w}\;=\;-5.3\times 10^{-2}+\;2.92\times 10^{-2}\times K–\;5.5\times 10^{-4}\times K^{2}\;+\;4.3\times 10^{-6}\times K^{3}.$$

To our knowledge, an equation of similar nature to Topp’s has not been developed for snow due to its heterogeneity and metamorphic changes over time. The studies discussed previously have all focused on developing their own empirical formulas (or using existing formulas) that relate snow dielectric permittivity and density to its LWC. For most mineral soils, Campbell Scientific states that Topp’s equation can be used to obtain LWC estimates using the CS650 dielectric permittivity measurements. However, they suggest a user-derived calibration for other media by describing the relationship between medium permittivity and liquid water content by a quadratic equation or a third order polynomial, much like Topp et al., depending on the number of data points and particular case. Data points should be spaced as evenly as practical over the expected range of LWC and include the wettest and driest expected values. In this paper, Equation (5) was used without any calibration due to the lack of in situ LWC measurements at the CREST-SAFE station. Because of this issue, and the fact that the equation presented by Topp et al. [33] was developed for mineral soils and does not take into account the effects of snow density on the dielectric constant and, indirectly, the LWC of the medium, empirical equations by Denoth et al. [21,22]:

(6) $$K_{r}\;=\;K-\;1-0.00192\times \rho_{snow}-0.44\times 10^{-6}\times {\rho_{snow}}^{2},$$

(7) $$\theta_{w}\;=\;-20.777+\;\sqrt{20.777^{2}+222.222\times K_{r}},$$

where $K_{r}$ is the dielectric constant reduced for variations in snow density $\rho_{snow}$, and Tiuri et al. [40]:

(8) $$K_{d}\;=\;1+1.7\times \rho_{d}+0.7\times {\rho_{d}}^{2},$$

(9) $$\theta_{w}=\;\frac{5}{72}\sqrt{28800\times \Delta K+7921}-89,$$

where $K_{d}$ is the dielectric constant under dry snow density $\rho_{d}$ conditions, and ΔK is the increase in permittivity due to wet snow conditions, were used as well. The empirical equations developed by Denoth et al. and Tiuri et al. have been validated in more recent snow studies [25,26,37,38,42]. Their results have shown that these empirical equations can, along with TDR, be used to provide LWC estimates within an accuracy of 1%–2% when compared to in situ observations. The comparison between LWC estimates using Equations (5), (7) and (9) was done in order to remediate the fact that actual in situ LWC measurements were missing at the station, as discussed in Section 1. The general idea is to cross-compare the three (3) LWC estimates obtained from these empirical equations with SNTHERM-simulated LWCs to see whether the model simulations are precise by comparing them with three different sources of LWC estimations.

### 3.5. Liquid Water Content Simulations Using SNTHERM

SNTHERM is a freely available, Fortran-written, one-dimensional snowpack physical model that simulates snowpack properties and was first released on 1989. Since its origin, it has been enhanced multiple times to improve its algorithms [48]. SNTHERM is energy and mass balance-driven. It has been used in several validation studies such as: snowpack spectral signature [49], snow melting processes [50], energy balances at regional scales, as well as discrete point scales, and for “under the canopy” snow [51,52]. A simplified version of the model is currently operational for snow mapping and forecasting in the United States [53,54,55] and Bosnia [56]. SNTHERM was developed using as foundation the mass and energy-balance snow model of Anderson [57]. The vertical water movement is based on the work done by Colbeck [58], in which the effective saturation of snow is a function of the current saturation level and the irreducible water saturation. Colbeck developed a flow model that accounts for multiple flow paths, transient ponding of water on ice layers, flow down distinct flow channels, and background flow simultaneously. He considered that the infiltration of liquid water through the ice layers is best described in a straightforward manner using his gravity flow theory [59], but the movement of the water in the flow channels is somewhat more complicated. Colbeck explained that flow channels are generated when water entering a dry, cold snow develops “fingers” that propagate ahead of the background flow leaving areas of cold, dry snow behind, and that the liquid water saturation in snow is too low for fingers to develop spontaneously simply from a heavier fluid (water) displacing a lighter fluid (air). Therefore, he hypothesized that the fingers are caused by the many crusts and ice layers normally present in highly stratified snow covers. This situation occurs many times in most snow covers because wind and melt crusts often mark the horizon between snow from separate storms. After flow fingers are established by the first movement of water through a snow cover, these original flow channels become preferential paths for future waves of infiltrating water due to the grain growth and permeability increase associated with the presence of liquid water in snow. However, on the scale of days or weeks leading to seasonal snow melt-off, the heterogeneous nature of the flow field is an important feature of most seasonal covers. The problems of multiple flow paths are complicated by the temporary ponding of water on individual ice layers. Hence, the volume of ponded water per unit width is calculated for a horizontal ice layer undergoing a steady balance of inflow and discharge to regularly spaced drains. He then concluded that, if the ice layer is sloping, if water is seeping simultaneously through the ice layer, or if transient effects are important, the volume of ponded water has to be adjusted accordingly. Thus, water in the snowpack propagates at a rate dependent on the flux ahead and behind the flow going directly through the ice layers [58]. However, the fluid flow model mentioned previously assumes horizontal homogeneity in the snow cover. In reality, seasonal snow covers that are undergoing freeze-thaw cycles, or that are subject to strong winds, develop crusts and ice layers, which complicate flow pattern. Thus, perforations arise in the crusts through which fingers of water flow at a much faster rate than through the crust itself [58]. Field observations by Marsh and Woo [60] of runoff rates from ripe snow in the Canadian Arctic showed that almost half the daily flow can be carried by fingers or flow channels that move ahead of the background front. They also developed a simulation model that incorporates the phenomenon of fingering [61]. Later, Schneebeli [62] showed that the location of fingers may not be stable in time and space. Additionally, more recent interpretations of preferential flow by Katsushima et al. [63] and Hirashima et al. [64] have demonstrated that flow fingers can develop even in isothermal conditions, and that ponding may allow to reach the necessary large concentrations of water. Within SNTHERM, snowpack layer densification is calculated based on three (3) main processes: destructive metamorphism or overburden compaction, constructive metamorphism or vapor movement and grain size change, and melt metamorphism or the gravitational water movement inside the snowpack [57,65,66,67]. The first two are merged into an overall compaction rate and the third metamorphism type is calculated based on the water balance. Destructive metamorphism is calculated as a function of snow viscosity [65,66]. Constructive metamorphism is a function of temperature and it is a process that has a faster rate when new snow density is greater than the density limit constant [57]. As stated by the Special Report 91-16 of the United States Army Corps of Engineers [48], and later on reported by [68], the SNTHERM numerical solution is obtained using a variable grid of snow layers, each layer being governed by heat and mass balance equations. The model uses a control volume numerical procedure [69] for spatial discretization that allows for the compaction of the snow. Lastly, a Crank-Nicholson central difference scheme is used to solve the partial differential equations in the time domain.

As mentioned in Section 2, the CREST-SAFE experiment is a long-term field campaign where meteorological variables and snowpack properties are measured. The measured meteorological variables at the station are: air temperature, solar radiation, relative humidity, and wind speed and direction. The only meteorological parameter that is not measured at CREST-SAFE (that is needed for simulation purposes) is precipitation. Therefore, it was obtained from the National Weather Service (NWS) Station named KCAR [70], located near (approximately 90 m away) the CREST-SAFE site at the Caribou Municipal Airport in Caribou, ME. The NWS uses the All Weather Precipitation Accumulation Gauge (AWPAG), instead of the traditional Heated Tipping Bucket (HTB) technology, in their Automated Surface Observing System (ASOS) stations to measure precipitation data. The AWPAG is essentially a weighing gauge where precipitation continuously accumulates within the collector, and as the weight increases, precipitation is recorded. The AWPAG has an 8-foot diameter outer shield to mitigate the wind effects on precipitation readings. Additionally, a transfer function provided by the World Meteorological Organization (WMO) is used to correct the precipitation measurements for undercatchment using daily average wind speed and maximum temperature observations performed by the ASOS station as well [71]. SNTHERM needs two precipitation parameters as input: precipitation water equivalent and precipitation type (rain or snow). Both variables were given to the model as obtained from the NWS precipitation records—these provide both the liquid and solid precipitation snowfall data. The precipitation deposition scheme to obtain the new snow height as solid precipitation accumulates over the snow surface is performed by SNTHERM based on its built-in algorithm [48]. Additional calibration parameters (i.e., irreducible water content for snow (0.017), density of new snow (73 kg/m^3^), density limit for compaction of snow (96 kg/m^3^), and the viscosity coefficient for overburden compaction (6.9 × 10^5^ kg·s/m^2^)) needed by SNTHERM to simulate the deposition scheme were established based on previous studies [48,57,65,66,67,72]. CREST-SAFE provides all of its meteorological data in an hourly time step via an automated routine [73], whereas the NWS provides precipitation data in 15-min time steps. Naturally, the NWS precipitation data was aggregated to hourly time intervals. Hence, by weather-forcing SNTHERM with the meteorological dataset at CREST-SAFE, layered hourly simulated snowpack properties (i.e., LWC, depth, grain size, density, temperature, and SWE) for the station were obtained for the period of this study.

It should be noted that Corona et al. [72] validated SNTHERM snowpack simulations (forced with CREST-SAFE in situ meteorological data) with three years (2010–2013) of CREST-SAFE in situ snowpack observations. More specifically, the SNTHERM evaluation was performed on properties such as: snow depth, SWE, density, temperature, and grain size, in addition to a layer-by-layer comparison of the snowpack properties. SNTHERM outputs showed high agreement with the observed data in properties like snow depth (R = 0.84), SWE (R = 0.77), density (R = 0.80), snow surface temperature (R = 0.98), and average snowpack temperature (R = 0.75). Conversely, the model was not very efficient when simulating properties like layer temperature (R = 0.54) and grain size (R = 0.60). Generally, SNTHERM appeared to simulate all snowpack properties closer to the snow surface better than those closer to the snow-ground interface. Additional studies by Lakhankar et al. [73] and Koivusalo and Heikinheimo [74] have also shown good agreement between various SNTHERM simulated snowpack properties and in situ observations. Lakhankar et al. demonstrated high agreement between bulk snow density (R = 0.97) and average grain size (R = 0.96) SNTHERM simulations and CREST-SAFE in situ observations. The differences in agreement between the studies by Corona et al. and Lakhankar et al. can be attributed to the fact that, in the former, the model vs. ground truth comparison was done at a layer-by-layer basis, whereas, for the latter, it was done with snowpack averages. Averaging snowpack properties will attenuate extreme (minimum and maximum) values in the dataset. Koivusalo and Heikinheimo compared SNTHERM simulations with in situ data from the Sodankylä Meteorological Observatory in Northern Finland. The results demonstrated high agreement between simulated and in situ snowpack properties such as: snow albedo, temperature, depth, SWE, and melt outflow. The proven good agreement between SNTHERM simulations and in situ snowpack properties, the CREST-SAFE-SNTHERM validation results by Corona et al., and the lack of in situ LWC measurements at the station led to the idea of using SNTHERM LWC simulations in this study.

While, to our knowledge, SNTHERM LWC simulations have not been validated directly, we understand that the high agreement between SNTHERM and in situ data for other snowpack parameters (i.e., depth, SWE, and density) shown in previous validation efforts provides sufficient evidence indicating that the SNTHERM LWC simulations (forced with in situ meteorological parameters) are, in fact, accurate. Firstly, because snow density has been described by snow hydrologists as the ratio between SWE and snow depth (indicating an intrinsic relationship between all three snow parameters), and, more importantly, because—in a simplistic approach—SNTHERM LWC simulations at each node (layer) $\theta_{wi}$ are calculated using the ratio between nodal liquid water bulk density $\rho_{wi}$ and nodal bulk snowpack density $\rho_{si}$:

(10) $$\theta_{wi}=\;\frac{\rho_{wi}}{\rho_{si}},$$

both of which are reliant on the SNTHERM calibration parameters (irreducible water content for snow, density of new snow, density limit for compaction of snow, and the viscosity coefficient for overburden compaction) that produced good validation agreement with the CREST-SAFE snow depth, SWE, and density observations, as stated previously. Each node containing a specific thickness; all amounting up to the total snowpack depth (n is the number of nodes/layers) and, consequently, LWC $\theta_{w}$ at its pertinent time step (every hour, in this study):

(11) $$\theta_{w}=\;\sum_{i=1}^{n}\theta_{wi}.$$

Thus, accurate SNTHERM LWC simulations will be highly dependent on the already proven accurate snow depth, SWE, and density simulations. However, the mass contribution of liquid water in snow is small, albeit important for hydrology. Hence, good snow density reproduction may be (at least partially) insufficient to infer that LWC and, equally important, liquid water location within the snowpack are also well reproduced. Instead, these issues might generate uncertainties in SNTHERM LWC simulations. For this reason, possible sources of model uncertainty, as related to the results in Section 4, will be discussed in Section 5.

It should be mentioned actual SNTHERM node/layer (not bulk) snow LWC values were compared with LWC estimates at single layer depths. The process consisted of finding the cumulative (sum of node/layer thicknesses) snow depth that would lead to the specific SWP sensor height, then the SNTHERM-simulated LWC at that node was extracted and used for comparison with LWC estimates by the three empirical formulas. SNTHERM bulk snow density computations were only discussed because these are part of its density simulation procedure.

Note: The irreducible water content for snow is the minimum amount of water that a layer of snow can hold; controlling evaporation and sublimation in the snowpack. Density of new snow is the assumed density for snow precipitation. Density limit for compaction of snow is the upper limit on destructive metamorphism compaction. Lastly, the viscosity coefficient controls the compaction rate of the snowpack due to overburden.

### 3.6. Evaluation Criteria

Three criteria were used in order to eliminate certain erroneous conclusions that could result from the use of one single evaluation criterion [75]. These were: the Root Mean Square Error (RMSE), Mean Absolute Error (MAE), and the correlation coefficient R. The selected evaluation criteria are widely used throughout the scientific community. Simultaneous analyses of these indexes will define the accuracy of the developed instrument. Generally, larger RMSE and lower R values are associated with more significant errors and poor agreement between the SWP and SNTHERM LWC estimates. Henceforth, there will be four (4) LWC annotations. These are: LWC_SNTHERM_, LWC_Topp_, LWC_Denoth_, LWC_Tiuri_; where each subscript makes reference to the model or equation these come from, as described in Section 3.4 and Section 3.5. The RMSE is computed as shown below:

(12) $$RMSE=\;\sqrt{\frac{\sum \text{​}{\left(Y_{obs}-Y_{sim}\right)}^{2}}{n}},$$

where $Y_{obs}$ is LWC_Topp_, LWC_Denoth_, or LWC_Tiuri_, $Y_{sim}$ is LWC_SNTHERM_, and n is the number of observations.

The MAE is calculated as follows:

(13) $$MAE\;\left(\%\right)=\;\frac{1}{n}\sum_{i=1}^{n}\left|Y_{sim}-\;Y_{exp}\right|,$$

where $Y_{exp}$ is LWC_Topp_, LWC_Denoth_, or LWC_Tiuri_ and $Y_{sim}$ is LWC_SNTHERM_.

While the correlation coefficient R is obtained as follows:

(14) $$R=\;\frac{\sum_{i=1}^{n}(X_{i}-\bar{X})\times (Y_{i}-\bar{Y})}{\sqrt{\sum_{i=1}^{n}{\left(X_{i}-\bar{X}\right)}^{2}}\times \sqrt{\sum_{i=1}^{n}{\left(Y_{i}-\bar{Y}\right)}^{2}}},$$

where $X_{i}$ is LWC_Topp_, LWC_Denoth_, or LWC_Tiuri_, $Y_{i}$ is LWC_SNTHERM_, $\bar{X}$ is the mean of the X dataset, and $\bar{Y}$ is the mean of the Y dataset.

## 4. Results

### 4.1. Evaluating the SWP’s Capability of Estimating LWC and Developing New Statistical Relationships for Different Snow Conditions

**Figure 3:**
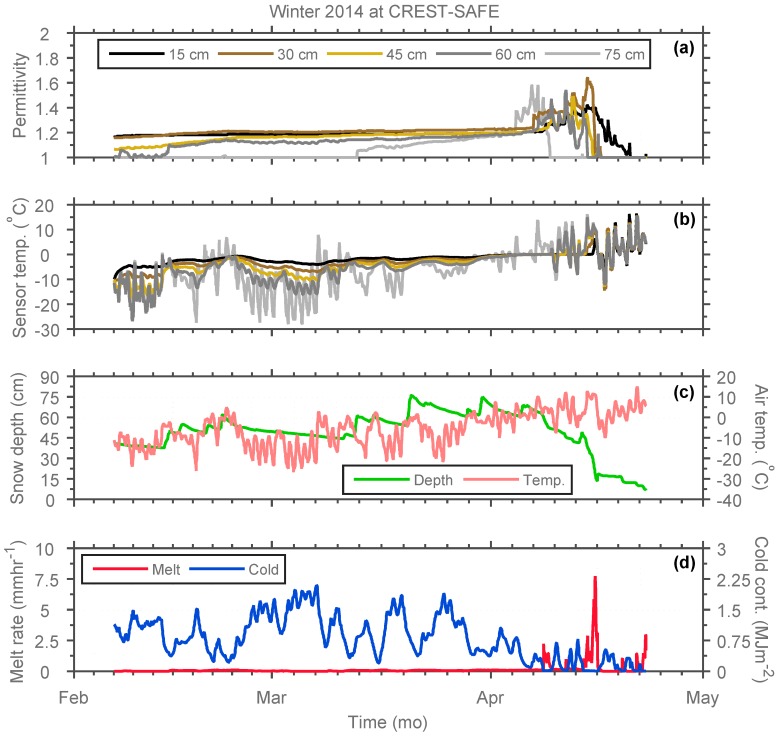
Snow Wetness Profiler snowpack dielectric permittivity (**a**) and temperature (**b**) measurements at different depths (15, 30, 45, 60 and 75 cm) above the soil surface at CREST-SAFE for winter (6 February–22 April) 2014; The third panel (**c**) illustrates snow depth (ultrasonic depth sensor) and near-surface air temperature (temperature and relative humidity probe) observations also collected at CREST-SAFE for the same period of time; The bottom panel (**d**) shows SNTHERM snowpack melt rate and cold content simulations obtained by weather-forcing the model with CREST-SAFE in situ meteorological data for the same time interval. All data are hourly.

The CREST-SAFE Snow Wetness Profiler in situ dielectric permittivity and snowpack temperature measurements at different depths (15, 30, 45, 60, and 75 cm) above the soil surface, along with snow depth and near-surface air temperature data from the station, and SNTHERM melt rate and cold content simulations for winter (6 February–22 April) 2014 are illustrated in Figure 3. It should be mentioned that the above isothermal temperatures (and respective dielectric permittivity values) recorded by the sensors are attributed to these being over the snow surface and exposed to the air temporarily. These observations are presented in Figure 3, but were eliminated from the analysis, as temperature in snow can only be lower than or equal to 0 °C.

As shown in Figure 3, the SWP is capable of reflecting changes in dielectric permittivity (Figure 3a) due to some melting events (e.g., 6–15 April 2014) (Figure 3c; changes in snowpack depth) and wetter periods with isothermal snowpack temperatures (most of April 2014) (Figure 3b). Hence, increases in snowpack temperature were consistent with those in dielectric permittivity, and vice versa. Snowpack temperature is affected because the seasonal snowpack grows in layered structure, causing heterogeneity, with each layer having different physical and mechanical characteristics. Then, the snowpack gets stratified because of successive snow events throughout the winter season. Hence, each snow event encounters a different set of meteorological parameters at the time of its occurrence and afterward. The snow continuously interacts with the environment and exchanges energy with the atmosphere above it and the ground below. These energy exchange processes set up the temperature distribution within the snowpack, which in turn is responsible for its metamorphic changes (in dielectric permittivity and LWC) with time [76,77,78]. Hence, the combination of above freezing temperatures during daytime with below freezing temperatures at night cause multiple freezing and melting events within the snowpack during the melting period. Additionally, daytime solar radiation causes snowmelt in the uppermost layer that produces higher dielectric permittivity and LWC in the superior layers of the snowpack. Furthermore, snowpack condition is considerably affected by air temperature. Previous studies [18,79,80] have shown that snow dielectric permittivity and LWC produce a significant and positive correlation with air temperature changes. These studies demonstrated that the average water content exponentially increased with the average air temperature and linearly increased with accumulated air temperature. This is confirmed by comparing Figure 3a–c, where air and snowpack temperature changes are significantly similar and linearly correlated, and dielectric permittivity changes are positively correlated with these two parameters.

However, LWC (and permittivity) changes can only be partially explained by temperature gradients. Hence, SNTHERM-simulated energy- (cold content) and mass-related (melt rate) snowpack parameters were included in Figure 3d to expand this analysis. The cold content of the snowpack is the energy required to bring the temperature of a dry snowpack to the temperature of melt (0 °C) [81]. This is a useful concept to interpret the delay between air temperature raising above 0 °C and actual melt outflow of a snowpack. Snowpack melt rate is associated with the amount of meltwater that will percolate through the snowpack and, ultimately, reach the soil. Generally, the meltwater produced at the surface percolates downwards through the snowpack. Because the speed of the percolation increases as the melt rate increases, water produced during the peak melt period overtakes water produced earlier in the day, such that the diurnal melt wave at successively deeper depths develops a sharp wave front. The time lag between the timing of peak surface melting and peak water output through the bottom of the snowpack increases with the depth of the snowpack [82]. Hence, e.g., a high cold content (and non-existent or low melt rate) can explain constant permittivity values during the pre-snowmelt period (no melt, just an increase in snowpack temperature). This is evidenced in Figure 3 by the mostly constant dielectric permittivity values associated with significantly low melt rate and high cold content from early-February to late-March. Conversely, increases in melt rate (combined with a shallower snowpack) and low cold content can be identified with higher dielectric permittivity values, and higher snowpack and near-surface air temperatures for the month of April.

According to Figure 3, during the pre-snowmelt periods, air temperature gradually increased and dielectric permittivity variations were relatively small due to high cold content and low melt rate. In this stage, the liquid water (at low melt rate) moves from the upper layer to the next layers and accumulates in the coarse snow layer [18,79,80]. Throughout the mid-snowmelt periods, the dielectric permittivity variation was drastic, as well as the changes in melt rate. The dielectric permittivity from the bottom layers (15, 30, and 45 cm) of the snowpack were smaller than those in the upper layers (60 and 75 cm). The variation was also smaller and more stable. During this period, liquid water is discharged from the snowpack [18,79,80]. Dielectric permittivity seemed to decrease during snowfall and the following one to two days. Lastly, during the late-snowmelt periods, the dielectric permittivity and temperature distribution and variation of every snow layer showed a uniform trend, and liquid water seemed to be moving to the next layer (at high melt rate). Lastly, it is easy to identify warmer melting snow temperatures (close to or at isothermal) consistent with higher dielectric permittivity (up to 1.6) values. Conversely, a low dielectric permittivity (~1) is congruent with either colder (<0 °C) snowpack temperatures or sensor exposure to air due to snow completely melting at that sensor height.

**Figure 4:**
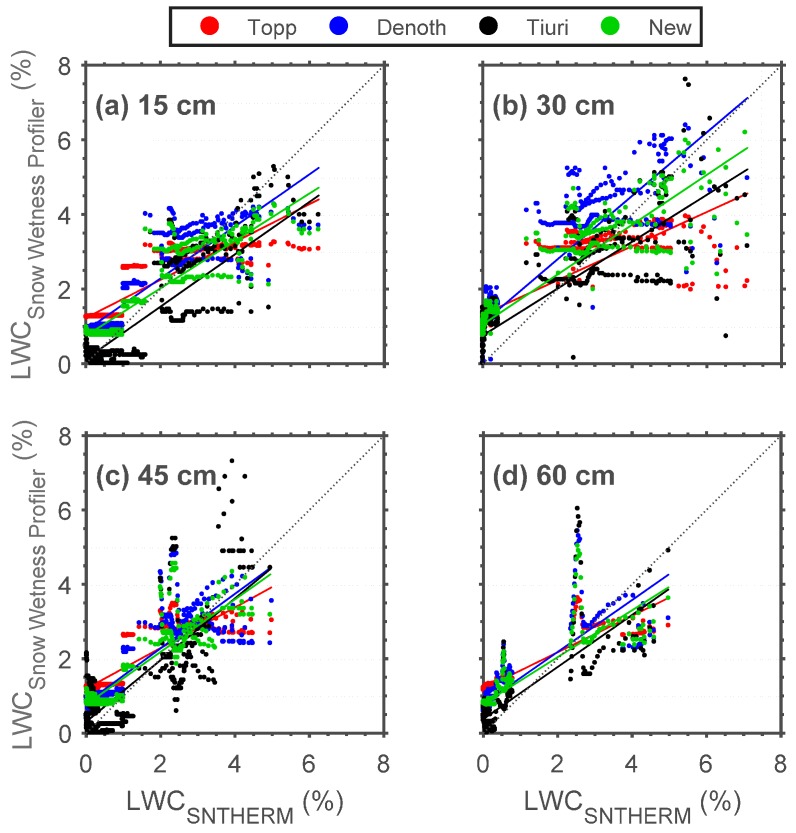
Snow Wetness Profiler (*y* axis) (estimated using Topp, Denoth, and Tiuri empirical formulas and developed statistical relationships) vs. SNTHERM (*x* axis) LWC scatter plots for different depths ((**a**) 15, (**b**) 30, (**c**) 45, and (**d**) 60 cm) above the soil surface at CREST-SAFE for winter (6 February–22 April) 2014.

The next step was to evaluate whether or not the SWP dielectric permittivity measurements can be translated into accurate LWC estimates. In Figure 4, Snow Wetness Profiler LWC (*y* axis) (LWC_Topp_, LWC_Denoth_, and LWC_Tiuri_) vs. LWC_SNTHERM_ (*x* axis) scatter plots for different depths (15, 30, 45, and 60 cm) above the soil surface at CREST-SAFE for winter (6 February–22 April) 2014 are illustrated. Table 3 describes the SWP performance by means of RMSE, MAE, and R values. In general, results indicate higher agreement between LWC_SNTHERM_ and LWC_Topp_, LWC_Denoth_, and LWC_Tiuri_ at the lower snowpack layers. This is congruent with the melting of the snow layers closer to the snow surface due to daytime solar radiation and warmer air temperatures. Results also positive correlations for all LWC estimates. Moreover, LWC_Denoth_ appears to better capture the LWC changes in the snowpack.

Because the results seem to be influenced by numerous low LWC estimates and the onset of the melt period (LWC > 2%) increases the permittivity and LWC scatter considerably, the LWC_SNTHERM_ values were divided into three snowpack categories/conditions: dry, moist, and wet. The idea being to provide insight as to whether or not the high agreement between LWC_SNTHERM_ and LWC_Topp_, LWC_Denoth_, and LWC_Tiuri_ is mostly due to dry snow conditions. The categories were selected based on a study by Fierz et al. [17]. However, we used three (instead of five) snow LWC categories. We decided to modify our snow LWC categories to fit specific LWC ranges because Fierz et al. defined their categories based mostly on manual (and physical) descriptions of the snow conditions (e.g., snow grains’ tendency to adhere to each other when pressed together, water not visible even at 10X magnification), but our study was executed remotely in automated fashion. This made it impossible for us to have specific knowledge on snow conditions like grain size and wetness. Instead, we followed the LWC ranges provided by Fierz et al. and made some adjustments. Because it seemed nearly impossible for SNTHERM to provide exactly 0% LWC, we decided to consider dry snow as snow with a LWC below 2%. Due to this modification, we proceeded to adjust moist snow from 0%–3% (Fierz et al.) to snow with a LWC between 2%–4%. Lastly, we defined wet snow as snow whose LWC is over 4%. Additionally, categories like ‘very wet’ and ‘slush’ were also considered to be (because they are) wet snow. The results (RMSE, MAE, and R) of the snow categorization comparison are shown in Table 4 by comparing LWC_SNTHERM_ with LWC_Topp_, LWC_Denoth_, and LWC_Tiuri_ for different snow conditions. Results indicate that there is higher agreement between LWC_SNTHERM_ and LWC_Topp_, LWC_Denoth_, and LWC_Tiuri_ whenever the snow is dry or wet, with low agreement for moist snow conditions. This clearly showed some limitations on all three empirical formulas that cannot capture the different behavioural patterns between dry and wet snow. 

**Figure 5:**
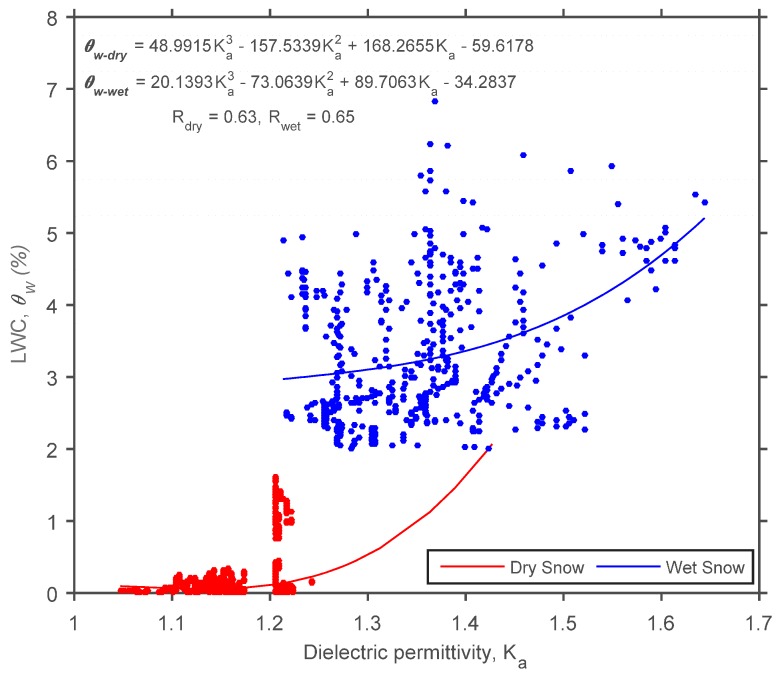
SNTHERM simulated LWC (*y* axis) vs. SWP dielectric permittivity (*x* axis) scatter plot for all depths (15, 30, 45, and 60 cm) above the soil surface combined at CREST-SAFE for winter (6 February–22 April) 2014. Third-degree polynomial regressions were found to be the best fit for dry (LWC < 2%) and wet (LWC ≥ 2%) snow conditions.

However, because SNTHERM LWC simulations appear to be precise when compared to the LWC estimates obtained using all formulas, this led to the notion that perhaps new statistical relationships between in situ snow dielectric permittivity and LWC simulations can be developed for different snowpack conditions. This way, even if unable to quantify the exact snow LWC, perhaps these new statistical relationships can detect the onset of melt, medium and maximum saturation of the snowpack. In Figure 5, all LWC_SNTHERM_ (*y* axis) were plotted against their respective observed SWP in situ dielectric permittivity (*x* axis) for winter (6 February–22 April) 2014 at CREST-SAFE. After analysing the results obtained in Figure 5, we came to the conclusion that only one threshold and two general snow behaviours were apparent. There seemed to be dry snow (<2%) for dielectric permittivity values below or equal to 1.2 and wet snow (≥2%) for dielectric permittivity values above 1.2. This was the reasoning behind the selection of this threshold. There was no clear statistical relationship between moist snow and dielectric permittivity. Hence, our general recommendation is for the user to create two statistical relationships based on this dielectric permittivity threshold value. Naturally, as demonstrated in Figure 5, the snow condition/LWC (dry or wet) threshold will be implicit. However, the snow LWC threshold has nothing to do (statistically) with the development of the statistical relationships. These were strictly created and validated using the dielectric permittivity threshold value of 1.2. Two cubic polynomial equations were found to be the best fit to describe the relationship between snow dielectric permittivity values and LWC for dry (<2% and permittivity ≤ 1.2) and wet (≥2% and permittivity >1.2) snow conditions. The statistical relationships are:

(15) $$\theta_{w-dry}\;=\;48.9915\times {K_{a}}^{3}-\;157.5339\times {K_{a}}^{2}+\;168.2655\times K_{a}-59.6178,\;(\text{perm.}\;\leq \;1.2)$$

(16) $$\theta_{w-wet}\;=\;20.1393\times {K_{a}}^{3}-\;73.0639\times {K_{a}}^{2}+\;89.7063\times K_{a}-34.2837,\;(\text{perm.}\;>\;1.2)$$

where $\theta_{w-dry}$ is the LWC of dry snow, $\theta_{w-wet}$ is the LWC of wet snow, and $K_{a}$ is the dielectric permittivity. The regression models exhibited correlations of R = 0.63 and R = 0.65 for dry and wet snow, respectively. The calibration results for these statistical relationships are shown in Figure 4.

Henceforth, the LWC obtained using the new statistical relationships presented will be referred to as: LWC_NEW_.

### 4.2. Validating SWP and new Statistical Relationships

This section intends to tackle on two subjects. The first being the validation of the SWP with CREST-SAFE 2015 data due to the fact that the sensors might have been partially exposed to the air in winter 2014. The second subject is the validation of the new statistical relationships shown in Section 4.1.

Dielectric permittivity can be difficult to calculate when the parallel rods are not completely covered by snow. If the rods are exposed to the air, this produces a major (decreasing) effect on the dielectric permittivity of the snow and can increase the error in LWC estimations [25]. While CREST-SAFE SWP 2014 data might have been affected by this phenomenon due to the removal of snow in order to install the instrument, 2015 data should not have been disturbed because no snow was removed around the SWP during that winter. As such, snow deposited around the SWP completely and the CS650 sensors were fully-covered in snow. To study the possible effects this might have had on SWP LWC estimates, Snow Wetness Profiler LWC_Topp_, LWC_Denoth_, LWC_Tiuri_ estimates were obtained and compared with LWC_SNTHERM_ for different depths (15, 30, 45 cm) above the soil surface at CREST-SAFE for winter (24 November 2014–29 January 2015) 2015. Table 5 shows the SWP performance by means of RMSE, MAE, and R values. Additionally, it demonstrates the performance of the new statistical relationships (LWC_NEW_) by comparing them with LWC_SNTHERM_ as well.

LWC_Topp_ and LWC_Denoth_ indicate general high agreement with LWC_SNTHERM_ at all snowpack layers, with LWC_Denoth_ showing better overall results again. This might be attributed to the fact that LWC_Denoth_ are not only reliant on dielectric permittivity, but are also snow density dependent. On the other hand, LWC_Tiuri_ exhibits significantly high errors. This might be credited to the first assumption the user has to make for dry snow density (200 kg/m^3^ in this study). Additionally, the dry snow density value used in the equation by Tiuri et al. was different from that calculated by SNTHERM in its subroutine (160 kg/m^3^). Though the difference is not particularly large, it is still a source of uncertainty when comparing LWC_Tiuri_ to LWC_SNTHERM_. Lastly, while it is known that dry snow density values will vary during a season and between winters, we decided to keep the same value for both winters to avoid additional uncertainties. It should also be noted that Equations (6) and (7) use bulk snow density values. The bulk snow density is the average density of the snowpack (obtained via in situ total snow depth and SWE values in this study), and not the snow density at each specific layer. Although the LWC estimates obtained with the equations by Denoth et al. displayed high agreement with SNTHERM LWC simulations, this could potentially present another source of error. Ideally, the layer snow density would be used in Equation (6) to obtain more accurate LWC estimates. Results display positive correlations for all LWC estimates. It should be mentioned that the CR3000 datalogger to which the SWP is connected stopped working after 29 January 2015 and had to be sent for repairment to its manufacturer. The SWP remained operational, but was not collecting data. From 24 November 2014 to 29 January 2015, the snow depth never reached 60 cm. This explains why only three snowpack layers (15, 30, and 45 cm) are shown in Table 5. There appears to be more variability between the LWC estimates by all empirical formulas when compared to winter 2014. However, these changes are not drastic enough (RMSE and MAE differences are between 1% and 2%, depending on the empirical formula used) to suggest that there was sensor overexposure to the air in 2014, nor that this exposure affected the dielectric permittivity values in any way. Furthermore, the MAE differences are smaller than those reported (2% or higher) by Lundberg [26] when the contact between the snow and sensor probes is influenced by air-gap formations. Hence, it is possible that these are interannual differences. 

LWC_NEW_ RMSE and MAE values range from 0.64–0.92 and 0.15–0.55, respectively. While R values varied from 0.77 to 0.82. All indicative of high agreement between LWC_NEW_ and LWC_SNTHERM_ and in agreement with previous studies [25,26,37,38]. In general, LWC_NEW_ appears to provide LWC estimates with better accuracy than the other empirical formulas. However, since it is more important to know whether or not the newly developed statistical relationships can estimate LWC accurately for dry and wet snow conditions, the LWC_SNTHERM_ values were divided into three (dry, moist, and wet) snowpack categories/conditions again, and the results were compared with LWC_Topp_, LWC_Denoth_, LWC_Tiuri_, and LWC_NEW_. The results (RMSE, MAE, and R) of the snow categorization comparison are shown in Table 6. Results indicate that there is higher agreement between LWC_SNTHERM_ and LWC_Topp_, LWC_Denoth_, and LWC_Tiuri_ whenever the snow is dry or wet, and lower agreement is exhibited for moist snow conditions. All three formulas yielded similar results. Additionally, interannual comparisons do not demonstrate significant changes in agreement between LWC_SNTHERM_ and LWC_Topp_, LWC_Denoth_, and LWC_Tiuri_ on a yearly basis. Lastly, when comparing LWC_SNTHERM_ with LWC_NEW_, results indicated improvements over all LWC estimations when using the new statistical relationships on different snow conditions. RMSE and MAE values ranged from 1.95–2.97 and 1.71–2.73, respectively, and R values fluctuated from 0.33 to 0.67. These results demonstrated smaller errors and better correlations when compared to the LWC estimates by all other empirical formulas for all three snowpack states. Furthermore, the LWC_NEW_ estimations displayed a must needed improvement over moist snow conditions; where dielectric permittivity and LWC measurements tend to be less stable.

Lastly, a brief (TDR dielectric permittivity) cross-comparison (MAE and relative error (RE)) between the two SWP rods at different snowpack heights above the soil surface is illustrated in Table 7. The general idea was to compare the two rods to—if the results were different—potentially estimate the influence of contact loss between the snow and sensor probes (air gaps). However, results indicate that both sensor arrays measured similar snow dielectric permittivity values at different snowpack heights. In reality, the distance between the two sensor rods is approximately 30–40 cm (quite a small margin to be considered conducive to significant spatial differences). Naturally, it seems logical then to think that both sensor rods faced (close to) identical conditions throughout the period of study. This was further evidenced by the online webcams we have at the site that allow us to have a real-time feed of the station at all times. Lastly, dielectric permittivity MAE/RE values for dry (permittivity ≤ 1.2) and wet (permittivity ≤ 1.2) snow conditions were 0.02/0.017 and 0.03/0.024, respectively. These results demonstrate that there is a possible heating (especially at heights closer to the snowpack surface due to solar radiation) of the probes that might create local snow melt around them; forming an air-gap. Hence, this local snow melting might not have happened for both sensor arrays at the same height. However, the MAE and RE values are not large enough to suggest that each sensor rod encountered significantly different conditions, such as contact loss between the snow and probes.

**Figure 6:**
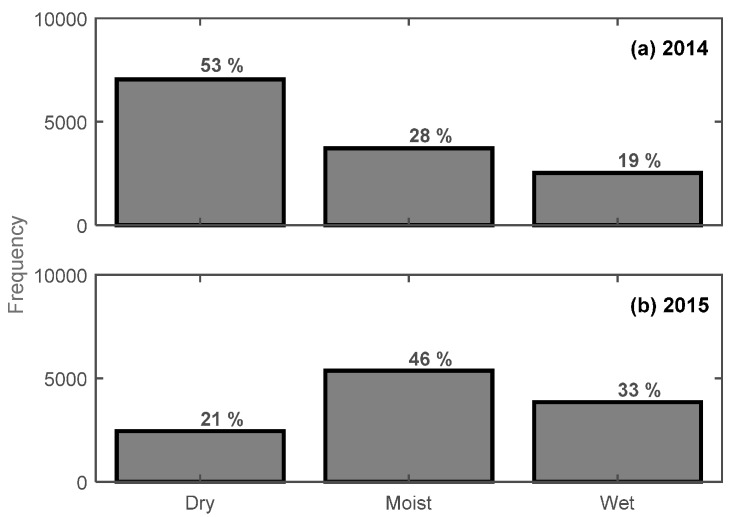
Frequency distribution for dry, moist, and wet snow conditions at CREST-SAFE for winters (**a**) 2014 and (**b**) 2015.

It should be noted that the frequency distribution (Figure 6) for the three snow categories/conditions was considerably different between years. This was expected due to the difference in observational periods between 2014 (February–April) and 2015 (November–January). At CREST-SAFE, the months of November and December are commonly associated with a “warm” (close to or at isothermal) snowpack and warmer air temperatures (leading to moist/wet snow) when compared to the months of January, February, and early-to-mid-March. February is a particularly cold month with a typically dry snowpack. While early-to-mid-April onwards commonly presents a warm, melting snowpack (associated with moist-to-wet snow). Nonetheless, different frequency distributions might have affected the results obtained using the new statistical relationships. Furthermore, because the results obtained using these new relationships demonstrated that estimating actual snow LWC values remains a difficult task (specifically for moist snow), we decided to evaluate the accuracy of these equations on a “hit-or-miss” basis using a confusion matrix. This way, we can at least tell whether the SWP can describe correctly the snow conditions (i.e., dry, moist, wet) even if it can’t provide the exact LWC value. Hence, the idea is to validate the capability of the new statistical relationships to “hit” the LWC_SNTHERM_ dry/moist/wet delimited thresholds (defined previously in this study) using CREST-SAFE 2015 data. 

**Figure 7:**
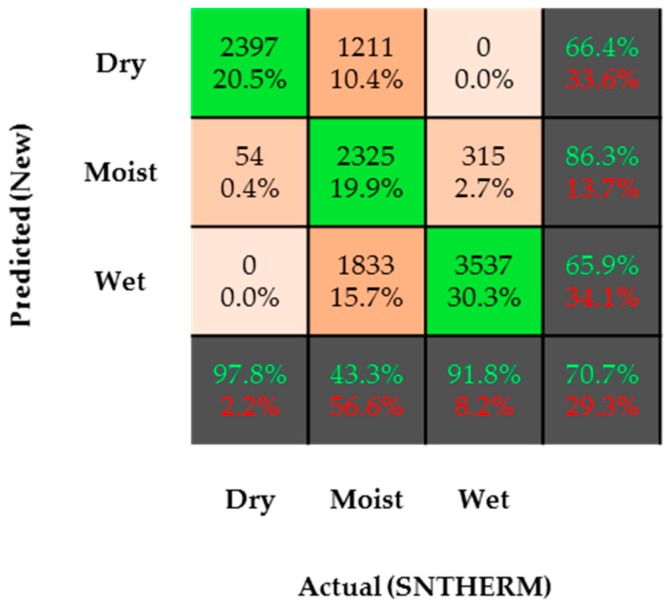
Confusion matrix (actual-SNTHERM vs. predicted-new statistical relationships) using three snow conditions (dry, moist, wet) as classes for CREST-SAFE 2015 validation data.

In general, a confusion matrix is a table that is often used to describe the performance of a model (equation or set of equations) on a set of test data (SNTHERM, in this case) for which the true values are known [83]. In this confusion matrix, there are three predicted (new statistical relationships) and actual (SNTHERM) classes (dry, moist, and wet snow). These make up a total of nine class comparisons. In Figure 7, numbers represent the amount of observations that make up a class comparison. 

Percentages define the percent value of the total number of observations for each class comparison. Green percent values inside the dark grey boxes describe the percentage of correct predictions for that particular row or column. Red percent values inside the dark grey boxes describe the percentage of incorrect predictions for that particular row or column. Results indicate that the new statistical relationships are capable of predicting the snow conditions with an accuracy of 70.7%. The true positive rates (actual and predicted match) for dry snow and wet snow were 97.8% and 91.8%, respectively. However, as expected, the true positive rate for moist snow was 43.3%. This demonstrates that the new statistical relationships were only correct (when compared to SNTHERM) half the time. However, when it comes to model precision, it is worth mentioning that whenever the new statistical relationships predicted moist snow, these predictions were correct 86.3% of the time. Model precision for dry and wet snow was 66.4% and 65.9%, respectively. These results demonstrate that the SWP was able to capture the changes in snow conditions, even when it was not able to provide exact LWC values.

## 5. Discussion

### 5.1. Advantages and Limitations

The SWP is inexpensive, and easy to install and assemble. This experimental setup is capable of detecting changes in LWC continuously and non-destructively over an entire melting period. This means changes in the LWC, such as daily melt-freeze cycles, can be traced with an hourly resolution. While it was not proven that the SWP can produce exact LWC measurements (especially for wet snow), it was demonstrated that it can recognize changes in the snowpack state (dry to wet conditions, and vice versa). Nonetheless, because the SWP provides the snowpack LWC and temperature profile, is makes it possible for snow scientists to gain insight as to how the water is seeping into the snowpack. However, all three empirical formulas (Equations (5), (7) and (9)) from previous literature [21,33,40] and the new statistical relationships (Equations (15) and (16)) developed in this study showed that capturing the variability in dielectric permittivity and LWC of wet snow accurately still remains a challenging task using TDR. These limitations can also be attributed to model uncertainties (Section 5.3), or be an intrinsic limitation of any instrument that lies in melting snow. While there was an apparent dielectric permittivity threshold value of 1.2 that seemed to separate dry and wet snow, it was still troublesome to provide exact LWC estimates for specific dielectric permittivity values under moist snow conditions. For this reason, it can only be inferred that dielectric permittivity values above 1.2 can produce LWCs between 2%–8%. This is shown in Figure 5, with the onset of the melting period and LWCs higher than 2%, the dielectric permittivity and LWC scatter increases considerably (1.2 < K_a_ < 1.5 and 2% < LWC < 5%). Thus, it seems more realistic to use the SWP to detect the onset of melt, medium, and maximum saturation of the snowpack, as demonstrated by the confusion matrix.

Lastly, the SWP can provide valuable information for hydrological applications, e.g., to detect the melt-onset with high temporal resolution. Particularly, if installed in large numbers at different locations; due to their cost efficiency and easy assembly. Sensor networks with large numbers of SWPs could be installed, monitoring on a large scale, e.g., melting processes of an entire hydrological catchment, or on a small scale, e.g., an avalanche prone slope, which is heterogeneously covered by snow.

### 5.2. Uncertainties When Estimating LWC Using TDR

In general, uncertainties in LWC estimates using TDR are dependent on several components. These are mainly snow density variations, temperature influences, and the contact between the probe rods and snow.

Aside from the trivial impacts inaccurate snow depth and SWE in situ data might have on snow density values (as discussed in Section 3.5), the dry snow density assumption as an input parameter for the formulas by Denoth et al. and Tiuri et al. may also be erroneous. Though the ultrasonic depth sensor and snow pillows were installed quite close to the SWP setup, snow depth at the SWP location can certainly deviate by a few centimeters. As such, an overestimation in snow depth leads to an underestimation of the LWC and vice versa; the higher this error is, the lower the snow depth is. A possible effect of snow depth (and density) variability could be reduced if the ultrasonic snow depth sensor would have been directly mounted above the SWP instrument, which was not physically possible. However, errors in snow depth larger than about ± 5 cm are rather unlikely at CREST-SAFE. A dry snow density value of 200 kg/m^3^ before the snow became wet was held constant over the entire melting period for the sake of the LWC determination using the snow density dependent formulas by Denoth et al. and Tiuri et al., as suggested by Mitterer et al. [15]. Nonetheless, it has been demonstrated that deviations in dry snow density (e.g., due to settling during the melting period) have little effect on the calculation of the LWC, especially when compared to those in wet snow density [84]. Lastly, one possible alternative to avoid uncertainties caused by the initial dry snow density assumption would be to conduct an independent density measurement right before the start of the experiment. On the other hand, wet snow density variations are quite impactful on LWC measurements using TDR. Lundberg [26] demonstrated that a change in density of 100 kg/m^3^ corresponds to a change in LWC of approximately 1%. A recent study [85] has shown that the higher the density, the higher the dielectric permittivity of snow. Also, the electrical performance of a snow-covered insulator string deteriorates as the density of the snow increases [86]. Lastly, bulk snow density was used in this study to provide some snow LWC estimates at different snowpack layers. While this is not ideal, it is common practice because it is quite hard to obtain in situ snow density values at each snowpack layer without manual measurements. This type of observations would hardly be continuous throughout 5–6 months of winter for obvious reasons. Naturally, this presents another source of uncertainty when trying to estimate snow LWC with a snow density dependent formula; especially when trying to obtain LWC estimates at specific locations within a snowpack knowing of its spatio-temporal snow density changes.

A study by Lu et al. [79] clearly demonstrated that the LWC of the snowpack is significantly affected by air temperature, which, naturally, affects snowpack temperature. In this study, the snow LWC showed a significant and positive correlation with the daily average, maximum, minimum, and accumulated air temperature. However, there was higher correlation between accumulated air temperature and LWC than that between average air temperature and LWC. Furthermore, the average LWC of a whole layer exponentially increased with the average air temperature and linearly increased with accumulated air temperature. The study also mentioned that the LWC scatter could only be partially attributed to different temperature indices, and that it was likewise partly caused by the mass balance of the snowpack. Techel and Pielmeier [18] even proved there to be specific snowpack temperature regimes where snow tends to be dry (snowpack temperature < −2 °C), probably moist (−2 °C ≤ snowpack temperature < 0.5 °C), or wet (−0.5 °C ≤ snowpack temperature < 0.5 °C). However, they also stated that prior knowledge of the snow grain size is needed to use these regimes in a snow study.

Lastly, it is important to make sure that there is contact between the probe rods and snow. As this lack of contact has been proven to yield inaccurate dielectric permittivity and LWC estimates [26]. Sensor exposure to the air will decrease dielectric permittivity and LWC measurements drastically. Furthermore, when probes are used in the field, sun radiation penetrates the uppermost l0 cm of the snow and may heat the probes and thus create local melt around probes forming an air-gap. These air-gaps will decrease the measured dielectric permittivity and, hence, the measured LWC. Lundberg [26] demonstrated that air-gap formations around the probes can decrease LWC measurements using TDR by more than 1% when the diameter of the hole increased from the diameter of the probe (5 mm) to 20 mm. The occurrence of air gaps can only be found during prolonged field measurements and are often unavoidable, since these happen naturally.

### 5.3. Possible Sources of Uncertainty in SNTHERM Simulations

In general, there are three characteristics that separate SNTHERM from most snow physical models (e.g., Biosphere–Atmosphere Transfer Scheme (BATS), Variable Infiltration Capacity (VIC) 2-L, National Operational Hydrologic Remote Sensing Center (NOHRSC) snow model (NSM), Lynch-Stieglitz snow model, Snow-Atmosphere-Soil Transfer (SAST), Community Land Model (CLM) snow model, Simplified Simple Biosphere model (SSiB), Noah). First, SNTHERM allows an unlimited number of layers to represent the vertical structure and thermal characteristics of the snowpack, whereas most snow models limit the number of snow layers to three. SNTHERM can therefore potentially resolve more detail in the snowpack profile, and a larger number of thinner layers can allow more accurate solution of vertical fluxes through the snowpack. Secondly, SNTHERM makes forward estimates by subdividing hourly meteorological inputs into smaller time steps, ranging between several seconds and several minutes, until convergence criteria for mass and energy fluxes in each layer have been satisfied. Long-term accuracy is maintained by ensuring the accuracy of each smaller time step. Lastly, SNTHERM uses the temperature and liquid water content of each layer to estimate the thermal conditions of the snowpack. Thus, SNTHERM accounts for the small fraction of liquid water that coexists in equilibrium with snow at temperatures less than 0 °C. However, these advantages (over other snow physical models) do not make SNTHERM uncertainty-free.

Over the years, SNTHERM has been used to simulate snow physical properties, validated with in situ snow observations, and compared with other snow models. Andreas et al. [87] validated SNTHERM with the snow, ice, and near-surface atmospheric processes on Ice Station Weddell (ISW). Ice Station Weddell produced over 2000 h of nearly continuous measurements in the atmospheric surface layer and in the snow and sea ice in the western Weddell Sea. Model results demonstrated high sensitivity to the density of newly fallen snow, which is estimated using a function dependent on wind speed and air temperature [88]. Furthermore, they used a new snow density value of 150 kg/m^3^ (twice the value we used in this study) because snow pit observations at ISW did not support values below this limit. Andreas et al. also reported misestimated heat fluxes in the snow due to errors in thermal conductivity. These errors were also attributed to new snow density. Additionally, SNTHERM, like most other one-dimensional snow models, assumes that the ice matrix and the interstitial air are at the same temperature and that the air is at rest. However, results by Andreas et al. suggested that the simulated near-surface temperatures and the measured snow-surface temperatures are often colder. Mote et al. [89] compared daily first-order SWE observations from five stations across the northern Great Plains with those estimated from passive microwave remotely-sensed data and SNTHERM. While they concluded that SNTHERM seems to adequately capture the seasonal mean SWE and seasonal cycle, there was a clear tendency for SNTHERM to underestimate SWE when compared to surface observations. This is especially significant given that surface observations are generally assumed to be underestimates of actual SWE. Most importantly, this is of specific significance to this study because SNTHERM SWE values affect bulk snow density and, ultimately, LWC simulations. Additionally, Mote et al. established that agreement between SNTHERM simulations and in situ observations increases substantially after applying an appropriate undercatch correction (also done in this study) to the precipitation data used to weather-force SNTHERM. While Feng et al. [90] studied the impacts of snow model complexity at three Cold Land Processes Field Experiment (CLPX) sites. They cross-compared simulations from the SSiB, Noah, VIC, CLM, and SNTHERM to field measurements from CLPX. When it comes to SNTHERM, early runoff was noted, owing to neglected water retention within the snowpack, potential to inaccurate LWC simulations. Furthermore, high snow albedo values in SNTHERM were shown to cause less solar radiation absorption, resulting in less energy for snowmelt. This could define the inaccuracy under wet conditions, which peaks at LWCs between 2%–4%, demonstrated in Section 4 when comparing SNTHERM-simulated with SWP-estimated LWC values. These values are usually measured at the beginning of the snowmelt season, which is probably the most difficult period for both in situ measurements and model simulations. Additionally, Feng et al. demonstrated that SNTHERM was unable to capture the observed runoff timing, even though the water storage and refreezing effects are included in the model physics. This implies that some uncertainty is associated with snow melting parameterization. They also reported that SNTHERM apparently overestimates snow density, which is likely a result of predicted excessive overburden within the snowpack, contributing to higher SWE values. Rutter et al. [53] compared four parameters (SWE, snow depth, average snowpack temperature, and snow surface temperature) estimated by the NSM with snow pit observations from five CLPX sites in Colorado and SNTHERM simulations. They stated that the methods used by SNTHERM to calculate thermal conditions and LWC in each layer of the snowpack are important, as they control the dominant mass flux (meltwater), which creates the SWE divergence that persists throughout ablation to create differences in melt-out times. These processes are greatly influenced by the irreducible water saturation (water withheld in the snowpack by capillary forces) and liquid water fraction. The former is a calibration parameter (0.04, in this study) that has to be established at the beginning of simulations, the latter is calculated by SNTHERM using a semi-empirical approach to calculate liquid water fractions within the snowpack as a function of snow temperature. Frankenstein et al. [91] carried out numerical experiments of snow accumulation and depletion, and surface energy fluxes over four CLPX sites in Colorado using SNTHERM and the Fast All-Season Soil Strength model (FASST). Their results showed that SNTHERM performs better when allowed to calculate the reflected solar radiation (done in this study), instead of being weather-forced by it. Shi et al. [92] investigated the lateral and vertical variability of snow stratigraphy by comparing the measured profiles of snow density, temperature, and grain size obtained during the Snow Science Traverse— Alaska Region (SnowSTAR2002) 1200-km transect from Nome to Barrow by comparing it to SNTHERM simulations. They explained that, because the SNTHERM soil model is simple and does not include many of the energy transport processes common to soils, errors of the snow temperature profiles increase closer to the snow–soil interface as a result of the simplified energy transport across the snow–soil interface, which only includes the thermal conduction and excludes the significant vapor diffusion. Additionally, SNTHERM snow density profiles failed to capture the hard and thin wind slabs because of the limitation of point model structure in representing the wind compaction effect.

Ultimately, even though SNTHERM has been proven to be one of the most complex and, consequently, accurate snow physical models, it remains a model nonetheless. As such, it will always remain a conceptualization of real snow physics and has to be treated accordingly. More importantly, the user should always be aware that the model outputs will always be reliant on accurate initial calibration and parametrization; as all study cases are and will be fundamentally different. Lastly, because the movement of liquid water in snow is a complex process, differences between SNTHERM and SWP LWC values might be due to both model errors and instrument inaccuracy.

### 5.4. Comparing the CS650 (and Its Precision) to Other Non-Destructive LWC-Measuring Instruments

When it comes to TDR snow LWC measurements, there are two widely-known instruments: the Denoth meter and Finnish Snow Fork [18]. The Denoth meter is a capacitance probe which measures an area of 13 × 9 cm^2^, operates at 27 MHz, and requires a separate density measurement to solve for the imaginary part of the permittivity. The Finnish Snow Fork samples an area of 6 × 2 cm^2^, operates at 1 GHz, and simultaneously measures both parts of the medium permittivity. Another commonly-used and -known TDR snow LWC measuring instrument is the Tektronix model 1502C [25,26]. The Tektronix model 1502C and its Tektronix PB30-58 balanced probes sample an area of 5 × 30 cm^2^, the system operates from DC to 1 GHz and also measures both parts of the medium permittivity. Other less used, yet still relevant TDR snow measurement instruments include: the automatic network analyzer (ANA) type R&S ZPV with tuner unit E3 (50 MHz to 1.5 GHz, 12.5 × 13.5 cm^2^) [93] and The Resonator (200 MHz to 1.4 GHz, 7 × 20 cm^2^) [94]. Additional non -TDR and -destructive related instruments commonly used to obtain snow LWC estimates are the SPA and upGPR. The SPA consists of two (2) SPA sensing bands with one installed horizontally 10 cm above the ground and the other installed at an angle (referred to as the sloping band), an impedance analyzer, an ultra-sonic snow depth sensor, and mounting accessories to assure proper tension of the SPA bands [29]. Each of the SPA bands sends frequencies into the snowpack and measures the complex impedance. The returned signals allow for the determination of liquid water, ice, and air percentages within the snowpack. The upGPR makes use of impulse radar (10 MHz to 2.6 GHZ) and bipolar antennas to estimate snowpack properties (e.g., LWC) [16]. Table 8 illustrates the accuracy and precision for snow LWC measurements for all the instruments mentioned in this section, as well as those of the CS650 reflectometer (for soil).

### 5.5. Comparing Results with Other Studies

In this study, average MAE and R values for LWC_Topp_, LWC_Denoth_, LWC_Tiuri_, LWC_NEW_ were 2.44, 0.31, 3.70, 0.37 and −0.67, 0.87, −0.53, and −0.79, respectively. As discussed previously, the empirical formula by Denoth et al. seems to provide the more accurate LWC estimates. Whereas the new statistical relationships show a slightly better performance over wet snow conditions. In general, when compared to the study by Stein et al. [25], the results are somewhat similar. Stein et al. reported average LWC MAE and R values of 1.3% and 0.81, respectively. While the investigation by Lundberg [26] reported LWC MAE values ranging from 1%–2%. Lastly, the results by Denoth et al. [21] displayed LWC MAE values of approximately 1%. However, it is difficult to compare the accuracy between studies. Because, although all of them used TDR to produce LWC estimates, the equipment used and general approach to each experiment were different. Stein et al. used different probe diameters and lengths. Additionally, the probes were aligned vertically (at the same height), not horizontally. Lundberg used snow control volumes produced at a laboratory, and Denoth et al. used 6 different instruments.

## 6. Conclusions

We presented an approach to continuously determine snow LWC at different snowpack layers with simple low-cost CS650 reflectometers using TDR. With this experimental setup, it was possible to estimate (with deficiency for moist snow) the snow LWC as a function of its dielectric permittivity. The Snow Wetness Profiler proof of concept demonstrates that it is possible to create an automated, continuous, non-invasive, and non-destructive way of conducting snow LWC and temperature profile observations to further improve our understanding of the interplay between the dielectric permittivity, temperature, and LWC of a snowpack.

The accuracy of the SWP for estimating LWC was validated using empirical formulas by Topp et al., Denoth et al., and Tiuri et al. These showed overall good agreement. However, all equations demonstrated an inability to provide accurate LWC estimates for wet snow. Hence, two (for dry and wet snow) new statistical (cubic polynomials) equations were developed between snow LWC and dielectric permittivity using CREST-SAFE in situ data. The equations displayed a better capability to capture the LWC changes in wet snow. Though, not good enough to be used to provide exact LWC estimates for specific dielectric permittivity values. Thus, at the moment, it seems more realistic to use the SWP to detect the onset of melt, medium, and maximum saturation of the snowpack. Uncertainties such as changes in snow density, snowpack and air temperature, and the possibility of partial sensor exposure to the air remain concerns when dealing with TDR field measurements. Nonetheless, the new statistical equations showed that formulas developed in previous literature have to be reconsidered because most studies provide one general applicable equation for LWC estimates, regardless of snow conditions. Dry and wet snow conditions should be treated separately. Furthermore, those studies that try to consider the differences between dry and wet snow require initial dry snow density or ice content assumptions. Lastly, it should be mentioned that, because these were developed using SNTHERM LWC values, the new statistical equations have to be used with caution. Naturally, it is recommended to use manual in situ LWC measurements and either improve the new statistical equations shown in this study, or develop alternate ones.

The main advantages of the SWP are its low cost and low power consumption, and that data analysis is neither time-consuming nor labor intensive. Additionally, the easy assembly makes it an appealing alternative for automated and continuous snow LWC measurements at a high temporal resolution without destroying the snow cover. Moreover, due to the small size of the instruments and the non-destructive measurement setup, these are virtually possible to install anywhere. In light of these preliminary results, the sensors and SWP show promise to become an alternative for in situ continuous and long-term snow LWC monitoring. However, additional work is needed to improve the accuracy of LWC estimations. As mentioned previously, the first recommendation is to use manual in situ LWC measurements to improve the new statistical equations. Secondly, these equations could be further improved if snow density values are incorporated as another independent variable. This was not done in this study because we think it is ideal to provide an equation (or equations) that is only dependent on one snow parameter. In reality, the reflectometers only provide dielectric permittivity values. Hence, if a snow density dependent equation was created, the users will need additional instrumentation to perform those measurements.

## Figures and Tables

**Table 1:** Instruments and environmental parameters observed at the CREST-SAFE research station in Caribou, ME, USA.

Parameter	Instrument	Automated (A) or Manual (M)	Accuracy ^1^	Sampling Interval
Meteorology				
Air temperature	Vaisala temp./RH probe	A	0.2 °C	3 min
Air humidity		A	0.7%	3 min
Wind speed	RM Young wind monitor (Alpine version)	A	0.3 m/s	3 min
Wind direction		A	3.0°	3 min
Snow/rain precipitation	Stand pipe station	A		3 min
Shortwave radiation	ukseflux 4-component net radiation sensor	A	1.0%	3 min
Longwave radiation		A	1.0%	3 min
Snow pack physical properties				
Snow depth	Ultrasonic depth sensor	A	1 cm	3 min
	Ruler	M	1 cm	2–3 days
Snow water equivalent	Snow pillow	A	N/A	3 min
	Gamma sensor	A	N/A	3 min
	Tube	M	1 mm	2–3 days
Snowpack temperature	Temp. probes (every 5 cm)	A	0.1 K	3 min
	FLIR infrared thermal imaging camera	M	0.2 K	2–3 days
Snow density vertical	Wedge cutter (every 10 cm)	M	N/A	2–3 days
Snow grain size vertical	Microscope (every 10 cm)	M	N/A	2–3 days
Snow liquid water content	Set of CS650 probes	A		1 h
Snow radiative/reflective properties				
Tb at 10 GHz (V/H)	Microwave radiometer	A	0.2 K	1 min
Tb at 18 GHz (V/H)	Microwave radiometer	A	0.2 K	1 min
Tb at 37 GHz (V/H)	Microwave radiometer	A	0.2 K	1 min
Tb at 89 GHz (V/H)	Microwave radiometer	A	0.2 K	1 min
Skin temperature	Apogee infrared radiometer	A	0.2 °C	3 min
Directional reflectance	CIMEL sunphotometer	A	0.01	1 h (daytime)
Other				
Soil moisture and temp.	Stevens Hydra probe	A		3 min
Aerosol optical depth	CIMEL sunphotometer	A	0.01	1 h (daytime)
Live images	Two web cameras	A	N/A	30 s

^1^ Accuracy of manual observations is an estimated value and corresponds to the typical accuracy for this type of measurement.

**Table 2:** CS650 operational specifications for soil temperature, dielectric permittivity, and LWC.

Parameter	CS650 Operational Specifications	
	Accuracy	Precision
Temperature	±0.5 °C	±0.02 °C
Dielectric permittivity	±(2% of reading + 0.6)	<0.02
Liquid water content	±3%	<0.05%

**Table 3:** Results from Snow Wetness Profiler LWC (estimated using Topp, Denoth, and Tiuri empirical formulas) vs. SNTHERM LWC comparison at different depths (15, 30, 45, and 60 cm) above the soil surface at CREST-SAFE for winter (6 February–22 April) 2014.

Layer Depth (cm)	Topp			Denoth			Tiuri		
	RMSE (%)	MAE (%)	R	RMSE (%)	MAE (%)	R	RMSE (%)	MAE (%)	R
15	1.76	1.70	0.89	0.96	0.86	0.89	1.88	1.80	0.89
30	1.93	1.87	0.69	1.24	1.12	0.68	1.91	1.81	0.46
45	1.78	1.75	0.74	0.88	0.79	0.74	1.98	1.92	0.74
60	1.88	1.86	0.62	0.87	0.79	0.62	2.13	2.06	0.62

**Table 4:** Results from Snow Wetness Profiler LWC (estimated using Topp, Denoth, and Tiuri empirical formulas) vs. SNTHERM LWC comparison for different snowpack conditions (dry, moist, and wet) at CREST-SAFE for winter (6 February–22 April) 2014.

LWC (%)	Topp			Denoth			Tiuri		
	RMSE (%)	MAE (%)	R	RMSE (%)	MAE (%)	R	RMSE (%)	MAE (%)	R
0–2 (dry)	2.03	1.78	0.54	0.85	0.84	0.54	2.11	1.81	0.55
2–4 (moist)	5.13	4.68	0.20	5.70	5.25	0.18	5.34	4.85	0.19
>4 (wet)	2.24	1.75	0.59	1.99	1.48	0.59	3.23	2.45	0.57

**Table 5:** Results from Snow Wetness Profiler LWC (estimated using Topp, Denoth, and Tiuri empirical formulas and new statistical relationships) vs. SNTHERM LWC comparison at different depths (15, 30, and 45 cm) above the soil surface at CREST-SAFE for winter (30 November 2014–29 January 2015) 2015.

Layer Depth (cm)	Topp			Denoth			Tiuri			New		
	RMSE (%)	MAE (%)	R	RMSE (%)	MAE (%)	R	RMSE (%)	MAE (%)	R	RMSE (%)	MAE (%)	R
15	2.49	2.45	0.65	1.62	0.49	0.87	3.66	3.58	0.53	0.64	0.15	0.82
30	2.48	2.45	0.67	1.07	0.20	0.85	3.74	3.72	0.54	0.71	0.42	0.78
45	2.42	2.41	0.69	0.95	0.23	0.88	3.80	3.80	0.52	0.92	0.55	0.77

**Table 6:** Results from Snow Wetness Profiler LWC (estimated using Topp, Denoth, and Tiuri empirical formulas and new statistical relationships) vs. SNTHERM LWC comparison for different snowpack conditions (dry, moist, and wet) at CREST-SAFE for winter (30 November 2014–29 January 2015) 2015.

LWC (%)	Topp			Denoth			Tiuri			New		
	RMSE (%)	MAE (%)	R	RMSE (%)	MAE (%)	R	RMSE (%)	MAE (%)	R	RMSE (%)	MAE (%)	R
0–2	2.33	2.05	0.47	1.03	0.93	0.51	2.17	1.83	0.46	1.95	1.74	0.67
2–4	4.83	4.31	0.18	5.15	4.90	0.20	5.27	4.92	0.15	2.97	2.73	0.33
>4	2.51	2.19	0.49	2.03	1.94	0.53	3.05	2.56	0.47	1.86	1.71	0.64

**Table 7:** TDR dielectric permittivity comparison (MAE and RE) between Snow Wetness Profiler rods at different depths (15, 30, 45, and 60 cm) above the soil surface at CREST-SAFE for winters 2014 and 2015.

Layer Depth (cm)	MAE	RE (%)
15	0.031	2.6
30	0.033	2.9
45	0.037	3.1
60	0.043	3.3

**Table 8:** Operational specifications for snow and soil LWC measurements for different LWC-measuring instruments.

Instrument	LWC Operational Specs	
	Accuracy (%)	Precision (%)
CS650 (soil)	±3	<0.05
Denoth meter (snow)	±2.5	<0.05
Finnish Snow Fork	±2.5	<0.05
Tektronix 1502C (snow)	±2.5	<0.05
ANA (snow)	±2	0.05
Resonator (snow)	±2	0.05
SPA	-	-
upGPR (snow)	±4–5	0.15

## Data Availability

The ground-observed datasets used in this study are available at the CREST-SAFE website: http://noaacrest.org//snow/. The sky cover dataset used in this investigation was obtained from the Caribou National Weather Service Regional Forecast Office website: http://www.weather.gov/car/.
